# Characterization of Disease Resistance Induced by a Pyrazolecarboxylic Acid Derivative in *Arabidopsis thaliana*

**DOI:** 10.3390/ijms24109037

**Published:** 2023-05-20

**Authors:** Michiko Yasuda, Moeka Fujita, Khamsalath Soudthedlath, Miyuki Kusajima, Hideki Takahashi, Tomoya Tanaka, Futo Narita, Tadao Asami, Akiko Maruyama-Nakashita, Hideo Nakashita

**Affiliations:** 1 Plant Acquired Immunity Research Unit, RIKEN Advanced Science Institute, Wako 351-0198, Japan; 2 Department of Bioscience and Biotechnology, Fukui Prefectural University, Fukui 910-1195, Japan; 3 Graduate School of Bioresource and Bioenvironmental Sciences, Kyushu University, Fukuoka 819-0395, Japan; 4 Graduate School of Agricultural and Life Sciences, The University of Tokyo, Tokyo 113-8657, Japan; 5 Graduate School of Agricultural Science, Tohoku University, Sendai 980-8572, Japan

**Keywords:** disease, phytohormones, plant activator, salicylic acid, systemic acquired resistance

## Abstract

Systemic acquired resistance (SAR) is a potent innate immunity system in plants that is induced through the salicylic acid (SA)-mediated signaling pathway. Here, we characterized 3-chloro-1-methyl-1*H*-pyrazole-5-carboxylic acid (CMPA) as an effective SAR inducer in *Arabidopsis*. The soil drench application of CMPA enhanced a broad range of disease resistance against the bacterial pathogen *Pseudomonas syringae* and fungal pathogens *Colletotrichum higginsianum* and *Botrytis cinerea* in *Arabidopsis*, whereas CMPA did not show antibacterial activity. Foliar spraying with CMPA induced the expression of SA-responsible genes such as *PR1*, *PR2* and *PR5*. The effects of CMPA on resistance against the bacterial pathogen and the expression of *PR* genes were observed in the SA biosynthesis mutant, however, while they were not observed in the SA-receptor-deficient *npr1* mutant. Thus, these findings indicate that CMPA induces SAR by triggering the downstream signaling of SA biosynthesis in the SA-mediated signaling pathway.

## 1. Introduction

Systemic acquired resistance (SAR) is a defense mechanism that is induced in the whole plant body after a hypersensitive response, including tissue necrosis caused by pathogens, through the salicylic acid (SA)-mediated signaling pathway [1,2,3]. As SAR is effective against a broad range of pathogens, it plays an important role in defending plants from pathogen attacks. SAR in *Arabidopsis thaliana* has been well characterized, and a set of pathogenesis-related (*PR*) genes have been identified as SAR marker genes [4,5,6,7]. Additionally, various types of SAR-deficient *Arabidopsis* mutants have been identified and used to investigate the mechanism of SAR. The SA biosynthesis-deficient mutant *sid2* contains a mutation in the *Isochorismate Synthase 1* (*ICS1*) gene [8]. The isochorismate generated in chloroplasts is transferred to the cytosol by Enhanced Disease Susceptibility 5 (EDS5) and converted into isochorismate-9-glutamate by AvrPphB Susceptible 3 (PBS3), which is then converted into SA by Enhanced Pseudomonas Susceptibility 1 (EPS1) [9,10]. Other mutant plants include members of the SA signal-deficient *Nonexpressor of PR Genes 1* (*npr1*) family, which have a mutation in the SA receptor protein [11,12,13,14], and NahG transgenic plants, which are unable to accumulate SA because they express the SA-degrading enzyme salicylate hydroxylase [6,7]. During SAR induction, the NPR1 proteins are reduced and transferred from the cytoplasm to the nucleus, in which an increase in the total glutathione (GSH) level takes part [15,16].

As SAR can protect the whole plant body against a broad spectrum of diseases, chemicals capable of inducing SAR have been exploited and used as plant activators to control economically important diseases in crop fields [17,18,19,20,21]. Such chemical activators exhibit several essential criteria for SAR inducers: induction of a broad spectrum of disease resistance, no or weak antibiotic activity against pathogens, and induction of SAR molecular markers such as *PR* gene expression. Some of these chemicals and their derivatives have been used to elucidate the mechanisms of SAR [15,22,23,24,25,26]. Benzo-(1,2,3)-thiadiazole-7-carbothioic acid S-methyl ester (BTH) stimulates downstream SA biosynthesis in the SAR signaling pathway [18,22], whereas 1,2-benzisothiazol-3(2*H*)-one1,1-dioxide (BIT) induces SAR by activating SA biosynthesis [24,27]. Similarly to BTH, 3-chloro-1-methyl-1H-pyrazole-5-carboxylic acid (INA) [16,28] and N-cyanomethyl-2-chloroisonicotinamide (NCI) [29,30,31] act at the site of the SA-mediated signaling pathway downstream of SA biosynthesis. In *Arabidopsis*, the induction of disease resistance by these chemicals requires a signaling pathway mediated by SA and the NPR1 proteins but not by jasmonic acid (JA) or ethylene (ET).

We previously reported that a pyrazolecarboxylic acid derivative, 3-chloro-1-methyl-1*H*-pyrazole-5-carboxylic acid (CMPA), induced disease resistance in rice against rice blast and bacterial blight [32,33], and it induced *PR* gene expression and disease resistance without SA accumulation in tobacco [34]. However, as the SA receptor-deficient mutant is not available in tobacco, the issue of whether CMPA activates the SA-mediated signaling pathway for SAR induction remains unelucidated. As the physicochemical properties of water-soluble CMPA are different from those of water-insoluble BTH, INA, and NCI, CMPA may activate pathways other than the SA–NPR1-mediated signaling pathway. Therefore, in this study, we aimed to determine how CMPA activates disease resistance using *Arabidopsis* plants.

## 2. Results

### 2.1. Induction of a Broad Range of Disease Resistance in Arabidopsis by CMPA

The ability of CMPA to enhance disease resistance in wild-type *Arabidopsis* (Col-0) was assessed using several virulent pathogens, including the hemi-biotrophic bacterial pathogen *Pseudomonas syringae* pv. *tomato* DC3000 (*Pst*), which causes bacterial speck; the hemi-biotrophic fungal pathogen *Colletotrichum higginsianum* (*Ch*), which causes anthracnose; the necrotrophic fungal pathogen *Botrytis cinerea* (*Bc*), which causes gray mold; and the viral pathogen cucumber mosaic virus (CMV). Soil drenching treatment with 0.25 mg/pot CMPA and foliar treatment with 1.25 mM did not cause any morphological changes or growth inhibition in plants, whereas foliar treatment with more than 6.25 mM CMPA resulted in tissue necrosis similar to a hypersensitive reaction (Supplementary Figure S1).

**Figure 1:**
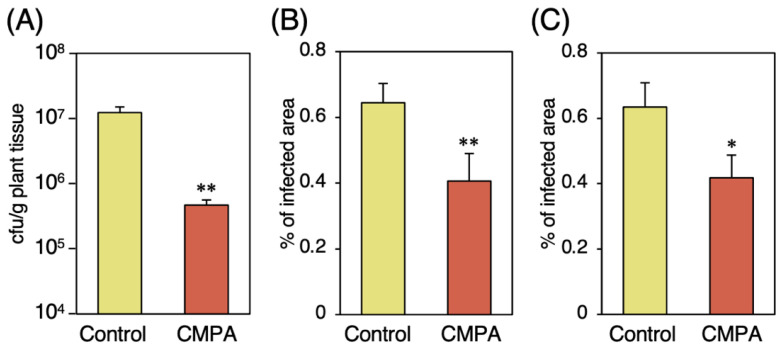
Effects of CMPA treatment on disease resistance in wild-type *Arabidopsis* plants. (**A**) Growth of *Pst* in CMPA-treated plants. Three-week-old Col-0 plants were treated with water (control) or CMPA (0.25 mg/pot) via the soil drenching method five days prior to inoculation with *Pst* (2 × 10^5^ cfu/mL). The population of *Pst* in the leaves was estimated three days after inoculation based on their growth on nutrient broth agar plates after homogenization in 10 mM MgCl_2_. Each experiment was performed with 10 samples prepared from three leaves. Values are shown as the means ± SE. (**B**,**C**) Lesion spreading of *Ch* (**B**) and *Bc* (**C**) in CMPA-treated plants. Three-week-old plants were treated with water (control) or CMPA (1.25 mM) via spraying five days prior to inoculation. Quantification of the lesion spreading of 30 independent virulence assays. Values are shown as the means ± SE. Significant differences between the control and CMPA-treated plants are indicated by asterisks. (Unpaired student’s *t*-test, * *p* < 0.05, ** *p* < 0.01.)

Susceptibility to *Pst* was estimated by measuring the bacterial growth in leaf tissues three days after the challenge inoculation. Treatment with 0.25 mg/pot CMPA by means of soil drenching five days prior to the challenge inoculation inhibited bacterial growth in the infected tissues relative to that in the water-treated control plants (96% reduction from the control) (Figure 1A). CMPA at concentrations of up to 200 µg/mL did not affect the growth rate of *Pst* in the liquid culture (Supplementary Table S1). These results indicated that CMPA enhanced resistance against *Pst* by activating the plant immune system.

Susceptibility to *Ch* was estimated using the area of necrotrophic lesions that appeared on the detached leaves two days after drop inoculation. Foliar treatment with 1.25 mM CMPA five days prior to the challenge inoculation reduced the ratio of the lesion area to infected leaf area compared to the untreated control plants (37% reduction from the control) (Figure 1B), indicating that CMPA enhanced resistance against *Ch* in *Arabidopsis*.

Disease resistance against *Bc* was estimated by measuring the size of the necrotrophic lesions that appeared on the infected leaves three days after drop inoculation. Foliar treatment with 1.25 mM CMPA was performed five days prior to the challenge inoculation. The ratio of spreading lesions to the whole infected leaves was significantly smaller in the CMPA-treated plants than in the untreated control plants (34% reduction from the control) (Figure 1C), indicating that CMPA enhanced resistance against *Bc* in *Arabidopsis*.

The effects of CMPA on CMV were estimated by detecting the viral coat protein in the plant tissues. Plants were foliar treated with 0.625 mM CMPA five days prior to the CMV inoculation. The results indicated that treatment with CMPA did not affect the viral multiplication and spread in the infected plants (Supplementary Figure S2).

### 2.2. Physiological Changes Associated with CMPA-Induced Resistance

**Figure 2:**
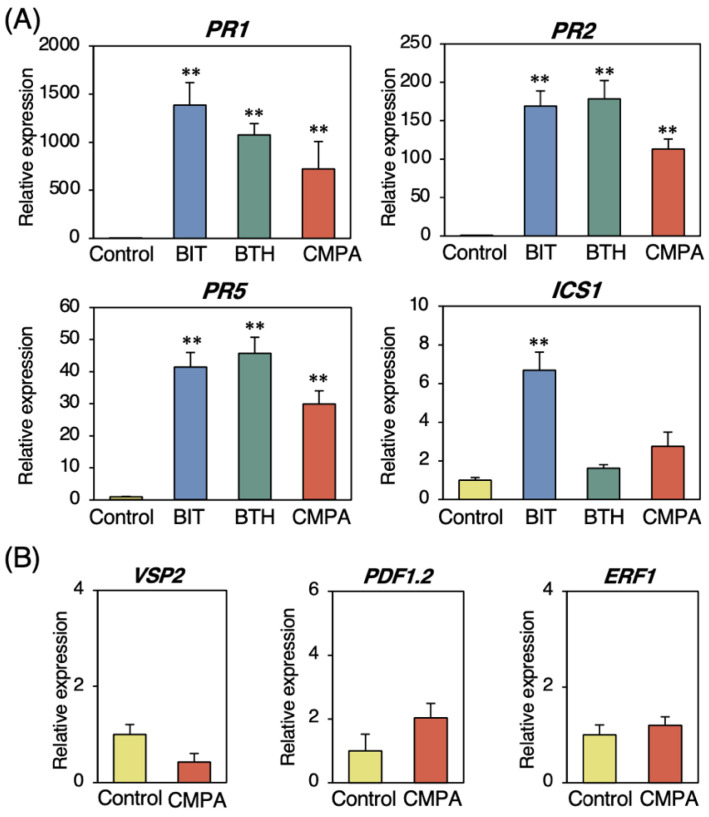
Expression of defense-related genes in wild-type *Arabidopsis* plants. Three-week-old plants were treated with water (control), BIT (1 mM), BTH (0.2 mM), or CMPA (0.5 mM) via spraying. The leaves were collected two days after treatment. An RT–PCR analysis was performed to evaluate the expression of (**A**) *PR1*, *PR2*, *PR5*, and *ICS1* and (**B**) *VSP2*, *PDF1.2*, and *ERF1*. The transcript levels were normalized to the expression of *UBQ2* measured in the same samples. Values are shown as the means ± SE (n = 6). Significant difference between the control and chemical-treated plants are indicated by asterisks. (Unpaired student’s *t*-test, ** *p* < 0.01.)

**Figure 3:**
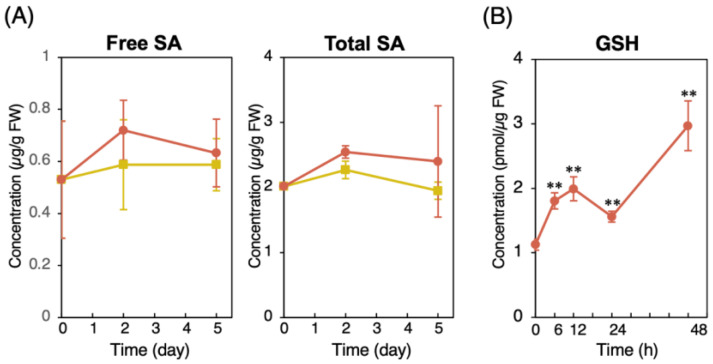
Accumulation of free and total salicylic acid and GSH in wild-type *Arabidopsis* plants treated with CMPA. (**A**) SA measurement in CMPA-treated leaves. Leaves were harvested two and five days after treatment with water (control) or CMPA (0.25 mg/pot) via soil drenching, and the free and total SA (free SA + SA glucoside) levels were quantified by means of HPLC. Values are shown as the means ± SD. Closed square (yellow), control; closed circle (red), CMPA. (**B**) GSH levels in leaves treated with CMPA. Leaves were harvested at 6, 12, 24, and 48 h after spraying with 0.125 mM CMPA, and the GSH levels were quantified by means of HPLC. Values are shown as the means ± SE. Significant difference between the control and chemical-treated plants are indicated by asterisks. (Unpaired student’s *t*-test, ** *p* < 0.01.)

To characterize the disease resistance induced by CMPA, we examined the physiological changes in the CMPA-treated wild-type plants. A gene expression analysis of SAR marker genes in the plants after two days of CMPA treatment was performed using the reverse transcription–quantitative polymerase chain reaction (RT–qPCR). The expression levels of *PR1*, *PR2*, and *PR5* were upregulated in the CMPA-treated plants compared with those in the untreated control plants (Figure 2A). The upregulation was similar to treatment with the SAR-inducing chemicals BIT and BTH (Figure 2A), indicating that CMPA activated the SA-mediated signaling pathway. The expression of the SA biosynthetic enzyme *ICS1* was unchanged with the CMPA and BTH treatments, whereas BIT upregulated its expression (Figure 2A). The expression levels of the JA-related genes *VSP2* and *PDF1.2* and the ET-related gene *ERF1* were not significantly different between the CMPA-treated plants and untreated control plants (Figure 2B), indicating that CMPA did not activate the JA- and ET-mediated signaling pathways. Endogenous SA accumulation in the leaves was examined two and five days after treatment with CMPA. The levels of free and total SA (free SA + SA glucoside) were not significantly different from those of the untreated control plants (Figure 3A). Increased endogenous GSH levels are important for SAR induction, and the level of GSH increased more than two-fold after two days of CMPA treatment (Figure 3B). These results indicated that CMPA induced SAR in *Arabidopsis* without SA accumulation. 

### 2.3. Effects of CMPA on SA-Deficient Mutant Plants

**Figure 4:**
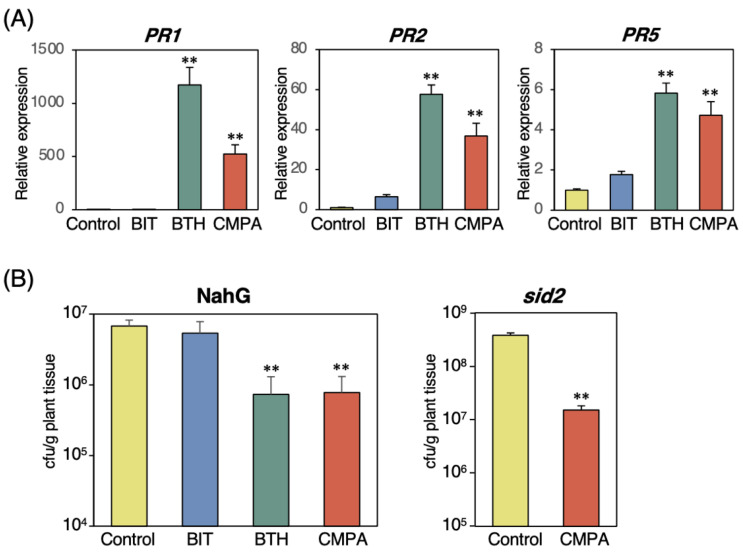
Effects of CMPA treatment on disease resistance and defense-related genes in SA-deficient *Arabidopsis* plants. (**A**) Expression levels of defense-related genes in the leaves of the *sid2* mutants treated with chemicals. Three-week-old plants were treated with water (control), BIT (1 mM), BTH (0.2 mM), or CMPA (0.5 mM) via spraying. The leaves were collected two days after treatment. An RT–qPCR analysis was performed to evaluate the expression of the *PR1*, *PR2*, and *PR5* genes. The transcript levels were normalized to the expression of *UBQ2* measured in the same samples. Values are shown as the means ± SE (n = 6). (Unpaired student’s *t*-test, ** *p* < 0.01.) (**B**) Growth of *Pst* in the SA-deficient mutant plants treated with chemicals. Three-week-old *NahG* and *sid2* plants were treated with water (control), BIT (0.5 mg/mL), BTH (0.1 mg/pot), or CMPA (0.25 mg/pot) via the soil drenching method five days prior to inoculation with *Pst*. The bacterial population in the leaves was estimated three days after inoculation. Each experiment was performed with 6 samples that were prepared from 3 leaves via homogenizing leaf disks in 10 mM MgCl_2_. Values are shown as the means ± SE (n = 6) (Unpaired student’s *t*-test, ** *p* < 0.01.)

To confirm whether SA biosynthesis was required for SAR induction by CMPA, we examined the effects of CMPA on the gene expression and disease resistance in *sid2* mutants defective in SA biosynthesis and NahG transgenic plants constitutively expressing SA hydroxylase, an SA-degrading enzyme. The expression levels of *PR1*, *PR2*, and *PR5* were significantly upregulated in the *sid2* mutant by CMPA and BTH but not by BIT (Figure 4A). CMPA treatment induced disease resistance against *Pst* in both the *sid2* and NahG plants (Figure 4B). These results indicated that SA biosynthesis was not required for the disease resistance and enhanced expression of SAR marker genes induced by CMPA.

### 2.4. Effects of CMPA on SA Receptor-Deficient Mutant Plants

**Figure 5:**
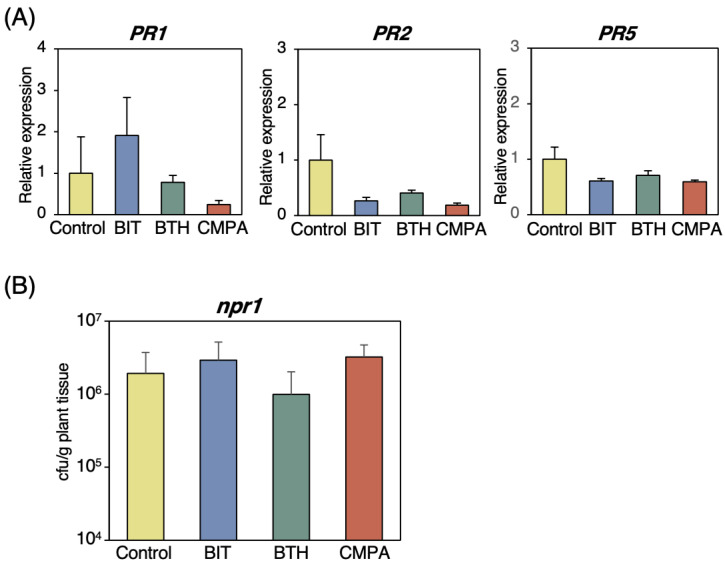
Effects of CMPA treatment on disease resistance and defense-related genes in the *Arabidopsis npr1* mutant. (**A**) Expression levels of *PR1*, *PR2*, and *PR5* in the leaves of the *npr1* mutant treated with chemicals. Three-week-old plants were treated with water (control), BIT (1 mM), BTH (0.2 mM), or CMPA (0.5 mM) via spraying. The leaves were collected two days after treatment. An RT–qPCR analysis was performed to evaluate the expression of the *PR1*, *PR2* and *PR5* genes. The transcript levels were normalized to the expression of *UBQ2* measured in the same samples. Values are shown as the means ±SE (n = 4). (**B**) Growth of *Pst* in the *npr1* mutant treated with chemicals. Three-week-old *npr1* plants were treated with water (control), BIT (0.5 mg/mL), BTH (0.1 mg/pot), or CMPA (0.25 mg/pot) via the soil drenching method five days prior to inoculation with *Pst*. The bacterial population in the leaves was estimated three days after inoculation. Each experiment was performed with 6 samples that were prepared from 3 leaves via homogenizing leaf disks in 10 mM MgCl_2_. Values are shown as the means ± SE (n = 6).

Analyses using wild-type and SA-deficient plants indicated that CMPA induced disease resistance and expression of SAR marker genes in an SA biosynthesis-independent manner. To determine whether SA-mediated signaling for *PR* gene expression was required for the CMPA-induced disease resistance, we examined the effects of CMPA on defense gene expression and disease resistance in *npr1* mutants defective in the SA receptor protein. The expression of *PR1*, *PR2*, and *PR5* did not increase in the CMPA-, BIT-, or BTH-treated plants, indicating that NPR1 was required for *PR* gene expression by CMPA (Figure 5A). The *Pst* inoculation assay showed that CMPA and the other SAR inducers did not enhance disease resistance in the *npr1* mutants (Figure 5B). These results indicated that SA-mediated signaling through the NPR1 protein was required for SAR induction by CMPA. 

## 3. Discussion

CMPA, without antimicrobial activity, enhanced disease resistance to a broad range of pathogens, including bacterial and fungal pathogens, and induced the expression of SAR marker genes in wild-type *Arabidopsis* plants, indicating that CMPA fulfills the criteria for a plant activator [18]. CMPA induced disease resistance and *PR* gene expression in the SA-deficient *sid2* mutant and NahG transgenic plant but not in the *npr1* mutant, indicating that CMPA induced SAR by activating SA-mediated signaling downstream of SA biosynthesis, as well as by BTH. This observation was supported by the *ICS1*, *VSP2*, *PDF1.2*, and *ERF1* expression or SA accumulation in the wild-type plants not being affected by CMPA.

Gray mold is an important disease requiring control because *Bc* causes disease in more than 200 plant species. Although *Bc* is a necrotrophic pathogen that does not induce SA-mediated signaling upon infection [35], the induction of resistance against *Bc* by exogenous SA or BTH has been reported in several plants, including *Arabidopsis*, tomato, and tobacco [36,37,38,39]. As CMPA is effective against *Bc* in *Arabidopsis*, it could also be effective in controlling gray mold in other plants, such as tomatoes and tobacco.

Although the physicochemical properties of water-soluble CMPA are different from those of water-insoluble BTH, INA, and NCI, their modes of action for resistance induction to *Pst* are similar. Whether CMPA acts directly on the NPR1 protein is unknown; however, this water-soluble plant activator might enable us to clarify the interaction between the plant activator and the NPR1 protein [13,14].

A carboxyl group at the five position of the pyrazole ring of CMPA is important for its SAR-inducing activity in rice [32]. Another SAR-inducing chemical with pyrazole moiety, 4-{3-[(3,5-dichloro-2-hydroxybenzylidene)amino]propyl}-4,5-dihydro-1*H*-pyrazol-5-one (BAPP), was revealed to act on the point downstream of SA biosynthesis in the SA-mediated signaling pathway in *Arabidopsis*, similarly to CMPA [16]. In contrast, BAPP contains a carbonyl group at the five position and a large substituent at the three position of the pyrazole ring. A recently developed plant activator, dichlobentiaox (3-(3,4-dichloro-1,2-thiazole-5-ilmethoxy)-1,2-benzothiazole-1,1-dioxide), contains benzoisothiazole and isothiazole rings, which are speculated to induce SAR in rice [40]. Thus, 1,2-azole moiety may be important for the activation of plant immunity. Analysis of the SAR induction activity of various types of 1,2-azole-type chemicals would be crucial to clarifying the regulatory mechanism of SA signal perception.

Treatment with CMPA did not result in resistance against CMV infection, whereas SAR was reportedly effective against CMV [41], suggesting that the mode of action of CMPA is different from that of other plant activators. When CMPA was applied to *Arabidopsis* leaves at high concentrations (>6.25 mM), tissue necrosis, similar to cell death during a hypersensitive response, was observed. This side effect was not observed at low CMPA treatment concentrations. Moreover, low CMPA concentrations did not affect the induction of resistance against bacterial and fungal pathogens. However, the combination of the invisible side effects, even at low concentrations, and the damage to the foliar surface due to rub inoculation with carborundum may explain why CMPA is not effective against CMV. As such a high-concentration solution cannot be prepared for water-insoluble BTH, INA or NCI, leaf necrosis is a specific characteristic of CMPA. Thus, this study revealed that CMPA is a novel type of plant activator, which stimulates the downstream of SA in the SA–NPR1-mediated pathway for SAR induction but induces a different spectrum of disease resistance. 

## 4. Materials and Methods

### 4.1. Plant Materials and Treatment

*Arabidopsis thaliana* plants were grown in sterilized potting soil Kumiai Nippi 1 (Nihon Hiryo, Tokyo, Japan) in pots (5 × 5 × 5 cm) inside a growth chamber under a 16 h:8 h light:dark cycle at 23 °C with 60% humidity. The three-week-old *Arabidopsis* plants were treated with CMPA, BIT, BTH, or water via soil drenching or via foliar spraying. 

### 4.2. Arabidopsis Pathogen Infection Assay

*Pst* was inoculated by dipping the plants in a bacterial solution (2 × 10^5^ colony-forming units/mL in 10 mM MgCl_2_) [24]. The leaves were harvested from the inoculated plants three days after inoculation and homogenized in 10 mM MgCl_2_. The homogenates were plated on nutrient broth agar containing rifampicin (50 mg/mL) at appropriate dilutions. After incubation for two days at 28 °C, the rifampicin-resistant bacterial colonies were counted. Bacterial growth was estimated using eight homogenates prepared from three leaves for each treatment. 

Drop inoculation of the fungal pathogens was performed by placing 3 µL of spore suspension of *Ch* (1 × 10^6^ spores/mL in water) or *Bc* (1 × 10^6^ spores/mL in 1/2 PDB medium) onto detached leaves that were placed on wet filter paper in a plastic box [42,43]. The box was sealed to maintain high humidity and incubated in the dark for three days at 25 °C. The areas of the lesions and whole leaves were measured using ImageJ software (ver 1.53). More than 30 leaves were used for each treatment, and the average ratio of the lesions to the whole leaf area was calculated to estimate the disease susceptibility.

CMV (20 µg/mL) was inoculated on a lower leaf of the plant via the rub inoculation method using carborundum [41]. The presence of viral coat protein in the plant tissues was detected via the tissue printing method at zero, three, and seven days after inoculation. The tissue printing method was performed by press blotting all the plants on filter papers, followed by probing the filters with a polyclonal anti-CMV-Y coat protein antibody.

### 4.3. Analysis of Gene Expression by RT–qPCR Analysis

The leaves were harvested 2 days after the application of the chemicals. The total RNA was extracted from the frozen leaf samples using Sepasol^®^-RNA I Super G (Nacalai Tesque Co., Ltd., Kyoto, Japan). The total RNA was used for the cDNA synthesis using the PrimeScript™ RT Reagent Kit with gDNA Eraser (Perfect Real Time) (Takara Bio Inc., Shiga, Japan). For the RT–qPCR, each cDNA sample was amplified using gene-specific primers (Supplementary Table S2) using TB Green^®^ Premix Ex Taq™ II (Tli RNaseH Plus) (Takara Bio Inc., Shiga, Japan) and a LightCycler^®^ 96 System (Roche Diagnostics K.K. Tokyo, Japan) [16]. Four to six biological replicates were used.

### 4.4. Extraction and Analysis of SA

Leaves were harvested from the treated plants two and five days post treatment with CMPA or water. The levels of SA and SAG were measured as previously described [24,44].

### 4.5. Extraction and Analysis of GSH

Leaf samples (approximately 100 mg for each sample) were harvested 0, 6, 12, 24 and 48 h after foliar treatment with CMPA. The extraction and measurement of GSH were performed as previously described [45,46]. Briefly, the GSH content in the leaf extract was determined via monobromobimane (Invitrogen) labeling of the thiol bases after the reduction of the extracts with dithiothreitol (Nacalai Tesque Co., Ltd., Japan). The labeled products were analyzed via HPLC with a fluorescence detector FP-920 (JASCO) using a TSKgel ODS-120T column (4.6 × 150 mm, Tosoh, Tokyo, Japan). The fluorescence of the thiol-bimane adducts was detected at 478 nm under excitation at 390 nm. The GSH standards were purchased from Nacalai Tesque.

## Data Availability

Data are available on request.

## Supplementary Materials

The following supporting information can be downloaded at https://www.mdpi.com/article/10.3390/ijms24109037/s1.
