# Supplementary material for: Characterization of Disease Resistance Induced by a Pyrazolecarboxylic Acid Derivative in Arabidopsis thaliana
Source: Int J Mol Sci. 2023 May 20;24(10):9037. doi: 10.3390/ijms24109037 (PMC10219097; doi:10.3390/ijms24109037)
Supplement: Supplementary file 1 [file ijms-24-09037-s001.zip › ijms-2165349-supplementary.pdf]

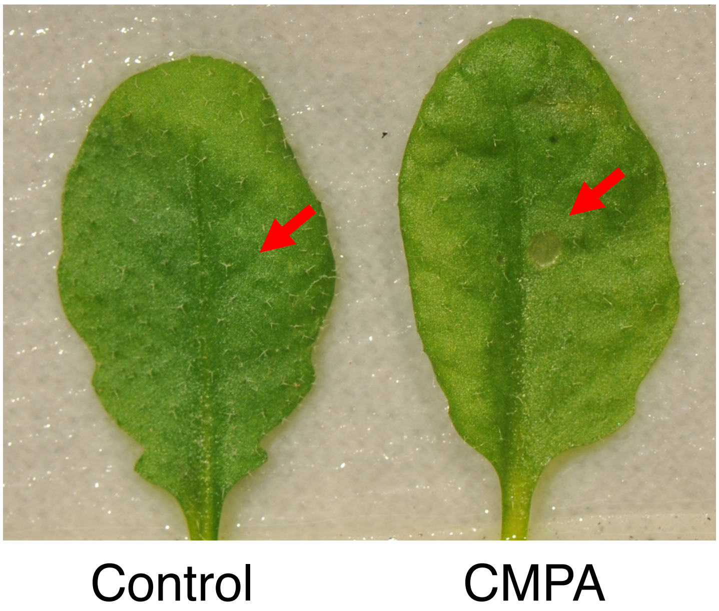

Figure S1. Cell death induced by treatment with CMPA.

Leaves of three-week-old wild-type plants were treated with 2 µl of water (Control) or 6.25 mM CMPA. Photographs were taken 18h after treatment. Arrows indicate the points of treatments.

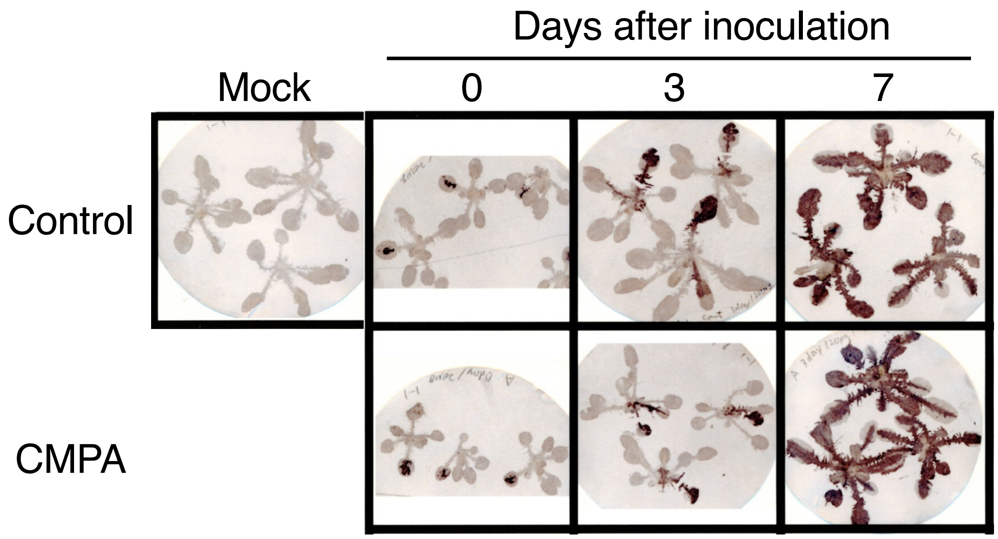

Figure S2. Effect of CMPA treatment on systemic spread of *Cucumber mosaic virus*-yellow strain (CMV-Y) in Arabidopsis wild-type (Col-0).

Three-week-old plants were treated with water (Control) or 0.625 mM CMPA by spraying 5 days prior to CMV inoculation. CMV (20μg/ml) was inoculated on a lower leaf of the plant by rub-inoculation method using carborundum. The presence of viral coat protein in plant tissues was detected by tissue printing method at 0, 3 and 7 days after inoculation. Tissue printing method was performed by press-blotting all the plants on filter papers, followed by probing the filters with a polyclonal anti-CMV-Y coat protein antibody.
The results indicate that viral multiplication and spread in Arabidopsis plants were not influenced by CMPA.

Table S1. Effects of CMPA on growth of *Pseudomonas syrinage* pv. *tomato* DC3000 in liquid nutritent broth.

CMPA concentration (μg/ml))	Bacterial growth (%)
1000	81.5
200	102.5
100	97.0
10	96.6
5	101.8

Pseudomonas syrinage pv. *tomato* DC3000 was cultured in liquid nutritent broth containing various concentration of CMPA for 48h at 28°C. Bactrerila growth was determined by measuring OD600 and presented as the relatve value to that of control without CMPA.

Table S2. List of primers used in this study

Gene	Name	Sequence (5'-3')
At2g14610	At_PR1_F	CGTCTTTGTAGCTCTTGTAGGTGCTC
	At_PR1_R	TCCTCGTGCCTGGTTGTGA
At3g57260	At_PR2_F	ACACGGCCAACATCCATCTAG
	At_PR2_R	TTGGTATGAGTACCCTGGATCGT
At1g75040	At_PR5_F	CGCTTATGACGACGAAACGA
	At_PR5_R	CGTGAGAGATAATTAAACCCGACTG
At1g74710	At_ICS1_F	GGGATAAGGGGTTCTCACAATAAG
	At_ICS1_R	AGGCTCGGCCCATTAACA
At2g36170	At_UBQ2_F	CAAGAGCTGTGAACTGCAGGA
	At_UBQ2_R	AAGGTTTGTGTCAGAACAATAGAGGAG
At5g44420	At_PDF1.2_F	TCATCATGGCTAAGTTTGCTTCC
	At_PDF1.2_F	ATTGCCGGTGCGTCGAA
At4g17500	At_ERF1_F	GAGCCGATACTCAGTGAGTCGA
	At_ERF1_F	GCTCTCGGTGAAGCAAGGATA
